# Plasma NfL and GFAP as Candidate Biomarkers of Disease Activity in NMOSD and MOGAD

**DOI:** 10.3390/medicina61101873

**Published:** 2025-10-18

**Authors:** Jarmila Szilasiová, Miriam Fedičová, Marianna Vitková, Zuzana Gdovinová, Jozef Szilasi, Pavol Mikula, Milan Maretta

**Affiliations:** 1 Department of Neurology, Medical Faculty, Pavol Jozef Šafárik University, Louis Pasteur University Hospital, 04011 Košice, Slovakia; miriam.fedicova@unlp.sk (M.F.); marianna.vitkova@upjs.sk (M.V.); zuzana.gdovinova@upjs.sk (Z.G.); milan.maretta@upjs.sk (M.M.); 2 Department of Ophthalmology, Medical Faculty, Pavol Jozef Šafárik University in Košice, Louis Pasteur University Hospital, 04011 Košice, Slovakia; jozef.szilasi@upjs.sk; 3 Department of Social and Behavioral Medicine, Medical Faculty, Pavol Jozef Šafárik University, 04011 Košice, Slovakia; pavol.mikula@upjs.sk

**Keywords:** neuromyelitis optica spectrum disorder (NMOSD), myelin oligodendrocyte glycoprotein antibody-associated disorder (MOGAD), biomarkers, glial fibrillary acidic protein (GFAP), neurofilament light chain (NfL)

## Abstract

*Background and Objectives*: Neuromyelitis optica spectrum disorder (NMOSD) and MOG antibody-associated disease (MOGAD) are distinct autoimmune demyelinating disorders of the central nervous system, characterized by different pathological and clinical features. Reliable biomarkers are essential for accurate diagnosis and monitoring of disease activity. Glial fibrillary acidic protein (GFAP) and neurofilament light chain (NfL) are promising candidates, reflecting astrocytic and axonal damage, respectively. *Materials and Methods*: To investigate the relationship between astroglial (GFAP) and neuronal (NfL) protein levels in the peripheral blood, 89 plasma samples were analyzed using Simoa immunoassays. The concentrations of pNfL and pGFAP were measured in three groups: AQP4-IgG-positive NMOSD patients (*n* = 18), MOGAD patients (*n* = 12), and healthy controls (HCs, *n* = 19). Statistical analyses assessed group differences, correlations, and the predictive value of biomarkers for disease activity. *Results*: Both NMOSD and MOGAD patients exhibited elevated pNfL compared with controls, indicating neuroaxonal injury. No significant differences in pNfL, pGFAP, or pGFAP/pNfL ratios were observed between patient groups. The pGFAP levels and the pGFAP/pNfL ratio were significantly higher in NMOSD patients, particularly during attacks, indicating prominent astrocyte damage. Correlations revealed associations between biomarker levels, disability, and disease duration. pNfL demonstrated high accuracy in predicting recent relapses (AUC = 0.906), whereas pGFAP showed moderate predictive capacity (AUC = 0.638). Elevated pNfL and pGFAP levels were associated with an increased likelihood of relapse within six months. *Conclusions*: Plasma NfL and GFAP are promising biomarkers for assessing tissue injury and disease activity in NMOSD and MOGAD. NfL predicts relapses, while GFAP primarily reflects astrocytic damage in NMOSD. Longitudinal studies are warranted to validate these biomarkers and establish clinical thresholds for disease management.

## 1. Introduction

Neuromyelitis optica spectrum disorder (NMOSD) and myelin oligodendrocyte glycoprotein antibody-associated disease (MOGAD) are distinct autoimmune demyelinating disorders of the central nervous system, primarily affecting the optic nerves and spinal cord [1]. NMOSD is a rare disease worldwide, predominantly affecting women [2]. MOGAD, also rare, was officially recognized as a separate entity from NMOSD in 2023 [3]. Despite differences in immunopathogenesis and clinical presentation, differentiating these conditions remains challenging, particularly in early or atypical cases. Accurate diagnosis, prognosis, and disease monitoring are essential for optimizing treatment; however, current clinical and radiological assessments often lack sufficient specificity and sensitivity. Consequently, there is an ongoing search for reliable biomarkers that reflect tissue damage and disease activity.

Glial fibrillary acidic protein (GFAP), an intermediate filament protein primarily expressed by astrocytes, has emerged as a promising biomarker of astrocytic injury and gliosis. Elevated GFAP levels in cerebrospinal fluid (CSF) and serum are associated with astrocytopathy, a hallmark feature of AQP4-IgG-positive NMOSD [4,5,6]. In NMOSD, an autoimmune attack against aquaporin-4 channels leads to astrocyte destruction, blood–brain barrier disruption, and secondary neuronal damage. Numerous studies have reported increased GFAP levels during acute attacks, with correlations observed between GFAP concentrations, MRI activity, and disability scores [5,6,7]. These findings suggest GFAP’s potential utility as a marker of active astrocytic injury and disease severity [8,9,10]. GFAP levels are highest during NMOSD relapse, particularly within the first 2 weeks, and decrease over the subsequent 1–3 months, often returning to baseline within approximately 3 months [11,12].

In MOGAD, unlike NMOSD, GFAP levels are less consistently elevated during attacks as MOGAD primarily involves demyelination rather than prominent astrocyte injury [10,11,13]. Despite these advances, the clinical use of GFAP in NMOSD and MOGAD remains under investigation. Its roles in monitoring disease activity, relapses, and treatment response require further validation, particularly regarding correlations with clinical and radiological parameters in MOGAD [5,13].

Another emerging biomarker is neurofilament light chain (NfL), a key component in axonal integrity. Elevated levels in CSF and serum reflect neuroaxonal damage, which is common in various CNS disorders. In NMOSD, secondary neuronal injury resulting from astrocyte loss and inflammation renders NfL a potential marker of axonal damage [14,15]. In MOGAD, which is mainly characterized by demyelination rather than astrocytopathy, the utility of NfL is still under investigation.

This study aimed to compare NfL and GFAP levels among AQP4-IgG-positive NMOSD patients, MOGAD patients, and healthy controls (HCs). We conducted an exploratory analysis examining the associations between these biomarkers and demographic factors (age and sex), as well as clinical variables (relapse occurrence and disability score). This approach seeks to determine whether NfL and GFAP can reflect underlying tissue damage and assist in clinical decision-making, particularly in monitoring disease activity related to attacks and persistent disability.

## 2. Materials and Methods

### 2.1. Patient Recruitment

This monocentric observational study was conducted at the Department of Neurology, Pavol Jozef Šafárik University Medical Faculty, and Louis Pasteur University Hospital in Košice from June 2023 to June 2025. Eighteen AQP4-IgG-positive NMOSD patients and twelve MOGAD patients, diagnosed according to current criteria, were included in the study.

The inclusion criteria were (1) a diagnosis of AQP4-IgG-positive NMOSD based on the IPND criteria [1] or a diagnosis of MOGAD based on the Banwell criteria [3]; (2) an age ≥ 18 years at inclusion; and (3) willingness to participate and provide written informed consent. Additionally, 19 healthy controls (HCs) were selected to closely match the age and sex distribution of the patient cohort.

For each patient, clinical and laboratory assessments were conducted annually from the first visit. Clinical status and disease activity over the preceding 12 months were evaluated, including Expanded Disability Status Scale (EDSS) scores, relapse occurrence, total number of relapses, and collection of blood samples for plasma NfL (pNfL) and plasma GFAP (pGFAP) measurements. If a patient experienced a clinical attack outside scheduled visits, an additional blood sample was obtained at the time of the attack to measure pNfL and pGFAP levels. Healthy controls underwent a single blood test for pNfL and pGFAP.

### 2.2. Study Methods

#### 2.2.1. Clinical Assessment

Attacks in NMOSD and MOGAD were defined according to standard criteria as acute or subacute clinical episodes characterized by neurological symptoms attributable to inflammation or demyelination of the central nervous system (CNS). They were supported by radiological and/or laboratory evidence and separated from previous attacks by a period of clinical stability [1,3].

Disease duration was defined as the interval from the onset of disease symptoms to the date of blood sample collection for pNfL and pGFAP analysis. Clinical data were collected retrospectively from medical records. The characteristics of the patient and control groups are presented in Table 1.

Throughout the study, patients received immunosuppressive therapies in accordance with established treatment guidelines. Assessments of clinical data and blood sampling were performed by different researchers, all of whom were blinded to each other’s results.

#### 2.2.2. NfL and GFAP Measurements

The plasma concentrations of GFAP and NfL were measured using SIMOA technology [16,17]. Venous blood samples (3 mL) were collected in vacutainer tubes containing an anticoagulant (sodium citrate) by the treating physicians and processed at room temperature within two hours. Samples were centrifuged at 4000 rpm for 10 min, and the collected plasma was aliquoted into polypropylene tubes and stored at −80 °C. All blood samples were pseudonymized and analyzed independently of clinical data.

Plasma NfL (pNfL) and GFAP concentrations were measured using the SIMOA^TM^ (Single Molecule Array) NF-Light Advantage Kit and Simoa GFAP Discovery Kit on the SIMOA HD-X Analyser (Quanterix, Lexington, MA, USA) following the manufacturer’s protocol. When analyzing the results in both NMOSD and MOGAD groups, pNfL and pGFAP samples were categorized as attack samples (within 3 months of the onset of an acute clinical attack) and remission samples (more than 3 months after the last clinical attack).

### 2.3. Statistical Analysis

The demographic, clinical, and laboratory data of patients are summarized as counts and percentages for categorical variables, means with standard deviations for normally distributed continuous variables, and medians with confidence intervals (CIs) for skewed data. The GFAP/NfL ratio was calculated based on pNfL and GFAP values. Intergroup differences (AQP4-IgG+ NMOSD vs. MOGAD vs. HCs) were assessed using standardized mean differences (SMDs), and group comparisons were performed with *t*-tests. For descriptive comparisons across groups, unadjusted *p* values were obtained from chi-square tests, a one-way ANOVA, or Kruskal–Wallis tests, as appropriate. Correlations between variables were evaluated using Pearson’s correlation coefficient when assumptions were met. Receiver operating characteristic (ROC) curve analysis was applied to evaluate the predictive performance of pNfL and pGFAP levels in relation to disease activity (relapses and EDSS scores). The area under the curve (AUC) with 95% confidence intervals (CIs) was calculated, and optimal cut-off values for the investigated parameters were determined. For longitudinal analyses, only complete datasets were included; no imputation of missing values was performed. Statistical significance was defined as *p* < 0.05. All statistical analyses were conducted using IBM SPSS Statistics, version 26.0.

### 2.4. Ethical Considerations

All patients provided written informed consent and agreed to the use of their anonymized data for research purposes. The study was approved by the Ethics Committee of L. Pasteur University Hospital in Košice on 15 May 2023 (approval no. 2023/EK/05024).

## 3. Results

The final cohort included 18 patients with AQP4-IgG+ NMOSD, 12 patients with MOGAD, and 19 healthy controls. Demographic, clinical, and biomarker data for patient groups and controls, including pNfL and pGFAP measurements, are presented in Table 1. The mean age of patients with NMOSD was 49.5 ± 10.0 years, whereas the MOGAD group had a mean age of 34.6 ± 13.2 years, and that of healthy controls was 39.0 ± 12.5 years. Of the 30 patients included, 6 (20%) were treated with steroids alone, 12 (40%) with steroids plus azathioprine, and 11 (37%) with rituximab, and 1 patient (3%) did not receive any immunosuppressive therapy (IMT).

Patients with AQP4-IgG+ NMOSD were older and more frequently female compared with both healthy controls and patients with MOGAD (Table 1). Disease duration and time since the last attack were longer, and EDSS scores were higher in the AQP4-IgG+ NMOSD group than in the MOGAD group. The total number of relapses did not differ significantly between patient groups.

In the subgroup including AQP4-IgG+ NMOSD patients, MOGAD patients, and healthy controls, where age, pNfL, and pGFAP were assessed, patients with AQP4-IgG+ NMOSD had a higher median pNfL concentration (6.9 pg/mL) than healthy controls (5.1 pg/mL, *p* = 0.048), as well as a higher median pGFAP concentration (169 pg/mL Vs. 133 pg/mL, *p* = 0.003).

Patients with MOGAD also showed a higher median pNfL concentration (6.4 pg/mL) compared with healthy controls (5.1 pg/mL, *p* = 0.018). The concentrations of pNfL and pGFAP and the GFAP/NfL ratio did not differ significantly between the two patient groups (Table 1).

Comparison of biomarker levels among NMOSD, MOGAD, and healthy controls showed the following results:

AQP4-IgG+ NMOSD Vs. Healthy Controls: pNfL levels were significantly elevated in NMOSD patients during attacks (*p* = 0.000), whereas no significant differences were observed during remission. Similarly, pGFAP levels were elevated during attacks (*p* = 0.004), returning to near-control values during remission. The pGFAP/pNfL ratio did not differ between NMOSD patients and healthy controls in either attack or remission.

MOGAD Vs. Healthy Controls: pNfL levels were increased during attacks (*p* = 0.005) and decreased during remission compared to controls (*p* = 0.005). pGFAP levels were elevated during attacks (*p* = 0.018), with no significant difference observed during remission. The pGFAP/pNfL ratio was lower during attacks (*p* = 0.000) but did not differ during remission.

AQP4-IgG+ NMOSD Vs. MOGAD No significant difference in pNfL levels was observed during attacks; however, pNfL was higher in NMOSD during remission (*p* = 0.034). pGFAP levels did not differ significantly between the two groups in either attack or remission. The pGFAP/pNfL ratio was higher in NMOSD during attacks (*p* = 0.016), with no significant difference during remission.

### 3.1. Correlations

#### 3.1.1. AQP4-IgG+ NMOSD

pNfL levels were significantly correlated with pGFAP levels (r = 0.643, *p* < 0.01). Elevated pNfL concentrations were associated with older age (r = 0.556, *p* < 0.01), higher EDSS scores (r = 0.582, *p* < 0.01), and shortened time since the last attack (r = −0.597, *p* < 0.01). Similarly, higher pGFAP levels were correlated with increased EDSS scores (r = 0.601, *p* < 0.01). The pGFAP/pNfL ratio was inversely correlated with age (r= −0.676, *p* < 0.01) and EDSS scores (r = −0.640, *p* < 0.01) but positively correlated with longer disease duration (r = 0.407, *p* < 0.05) (Table 2). 

#### 3.1.2. MOGAD

pNfL levels were significantly correlated with pGFAP levels (r = 0.702, *p* < 0.01), and higher pNfL concentrations were associated with older age (r = 0.596, *p* < 0.01), shorter time since the last attack (r = −0.513, *p* < 0.01). Elevated pGFAP concentrations were associated with higher EDSS scores (r = 0.452, *p* < 0.01). The pGFAP/pNfL ratio was inversely correlated with age (r = −0.496, *p* < 0.05) and positively correlated with a greater number of relapses (r = 0.563, *p* < 0.01) and longer time since the last attack (r = 0.79, *p* < 0.01).

### 3.2. Predictive Models

#### 3.2.1. Receiver Operating Characteristic (ROC) Curves for Biomarker Prediction of Relapse Absence Within the Past 6 Months

**Figure 1:**
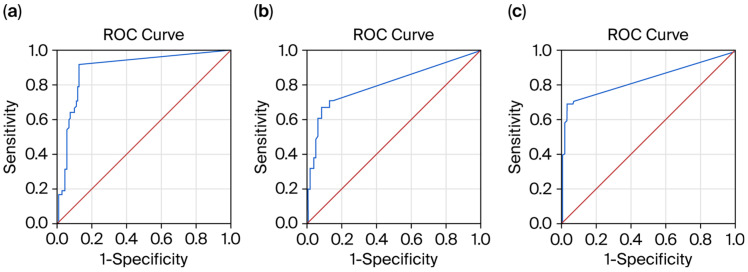
Receiver operating characteristic (ROC) curves for biomarker prediction of relapse absence within the past six months: (**a**) pNfL; (**b**) pGFAP; and (**c**) pGFAP/pNfL ratio. Blue line—ROC curve representing biomarker prediction; red line—reference line.

The ROC curve analysis demonstrated that the pNfL level was a significant predictor of relapse absence within the past 6 months (AUC = 0.906; 95% CI, 0.844–0.968; *p* < 0.01). The sensitivity and specificity of the predictive model were 96.6% and 84%, respectively (Figure 1a). Lower pNfL values indicated stronger evidence for relapse absence within the past 6 months, with a cut-off level of 5.8 pg/mL.

The pGFAP level showed a weak predictive value for relapse absence within the past 6 months (AUC = 0.638; 95% CI, 0.519–0.758; *p* < 0.01). The sensitivity and specificity of the predictive model were 64.5% and 61.2%, respectively (Figure 1b). Lower pGFAP values indicated stronger evidence for relapse absence within the past 6 months, with a cut-off level of 82.5 pg/mL for pGFAP.

The pGFAP/pNfL ratio demonstrated good predictive performance for relapse absence within the past 6 months (AUC = 0.814; 95% CI, 0.726–0.901; *p* < 0.01). The sensitivity and specificity of the predictive model were 76.6% and 74%, respectively, with a cut-off level of 5.6 (Figure 1c).

#### 3.2.2. Receiver Operating Characteristic (ROC) Curves for Biomarker Prediction of Relapse Absence Within the Past 12 Months

**Figure 2:**
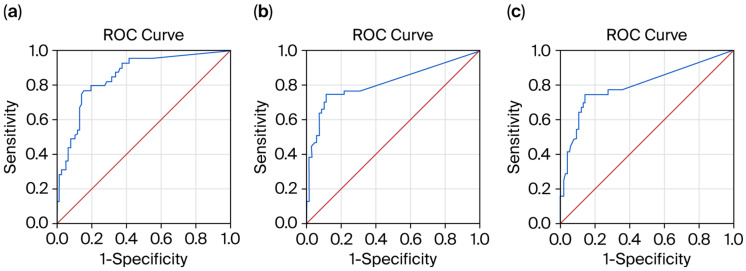
Receiver operating characteristic (ROC) curves for biomarker prediction of relapse absence within the past 12 months: (**a**) pNfL; (**b**) pGFAP; and (**c**) pGFAP/pNfL ratio. Blue line—ROC curve representing biomarker prediction; red line—reference line.

The ROC curve analysis demonstrated that the pNfL level was a significant predictor of relapse absence within the past 12 months (AUC = 0.891; 95% CI, 0.830–0.952; *p* < 0.01). The sensitivity and specificity of the predictive model were 91.7% and 98.1%, respectively (Figure 2a). Lower pNfL values indicated stronger evidence for relapse absence within the past 12 months, with a cut-off level of 4.4 pg/mL.

The pGFAP level showed a weak predictive value for relapse absence within the past 12 months (AUC = 0.629; 95% CI, 0.521–0.737; *p* < 0.01). The sensitivity and specificity of the predictive model were 65% and 87%, respectively (Figure 2b). Lower pGFAP values indicated stronger evidence for relapse absence within the past 12 months, with a cut-off level of 94.6 pg/mL.

The pGFAP/pNfL ratio showed a moderate predictive value for relapse absence within the past 12 months (AUC = 0.797; 95% CI, 0.701–0.892; *p* < 0.01). The sensitivity and specificity of the predictive model were 77% and 67.5%, respectively (Figure 2c). Lower pGFAP/pNfL ratios indicated stronger evidence for relapse absence within the past 12 months, with a cut-off level of 5.2.

Among the tested biomarkers, pNfL was identified as the best predictor of relapse absence within both the past 6 and 12 months

## 4. Discussion

This study aimed to evaluate the potential utility of glial fibrillary acidic protein (GFAP) and neurofilament light chain (NfL) as biomarkers of disease activity in AQP4-IgG-positive neuromyelitis optica spectrum disorder (NMOSD) and MOG antibody-associated disease (MOGAD). We compared plasma NfL and GFAP levels in patients with these conditions and in healthy controls (HCs). Our findings both corroborate and extend prior research, which has reported similar or contrasting patterns.

### 4.1. Neurofilaments

Mean plasma NfL levels were significantly elevated in both NMOSD and MOGAD patients compared to HCs, consistent with previous studies [10,18]. In both disorders, pNfL levels increased during attacks relative to remission, in line with the existing literature [10,18]. However, Kim et al. reported comparable NfL levels during relapse and remission in MOGAD [11]. During attacks, pNfL levels were approximately twice as high in NMOSD and three times higher in MOGAD compared with remission. However, pNfL levels during remission remained higher in NMOSD than in MOGAD (5.2 vs. 3.6 pg/mL). These findings suggest that neuroaxonal injury is associated with relapses, with a more pronounced pNfL elevation followed by a decline in MOGAD, whereas NMOSD exhibits persistently elevated levels during remission, potentially reflecting residual structural damage or ongoing neurodegeneration. Interestingly, pNfL levels during remission in NMOSD approximated those of healthy controls and were even lower in MOGAD, possibly reflecting therapeutic effects. NfL levels correlated with age, reflecting age-related neurodegeneration, and with time since the last relapse in both conditions. A significant association with disability scores (EDSS) was observed only in NMOSD, consistent with the findings of Vanatabe et al. [8]. Some studies also reported correlations between NfL and disability in MOGAD [11,18]. Our ROC analysis further supports NfL as a biomarker of disease activity, pNfL was identified as the best predictor of relapse absence within both the past 6 and 12 months demonstrating high predictive accuracy (AUC = 0.903). Our results are exploratory in nature; therefore, the observed predictive performance of NfL for relapse activity should be interpreted with caution. The small sample size and single-center setting may lead to overestimation of accuracy, and further research is needed to confirm these findings. While AQP4-IgG and MOG-IgG titers tend to increase during relapses, routine monitoring is not currently standard practice [13]. Our data reinforce the potential of pNfL as an accessible marker of tissue damage and disease activity, particularly during acute phases. Our findings regarding the MOGAD cohort are consistent with previous reports [18,19], indicating that pNfL reflects neuroaxonal injury during relapse rather than overall disease activity.

### 4.2. Glial Fibrillary Acidic Protein

Consistent with previous studies, pGFAP levels were elevated in NMOSD patients during attacks, being approximately two-fold higher than during remission [5,6,8,10,11]. This underscores the utility of pGFAP as a sensitive marker of astrocyte injury in NMOSD, particularly during acute episodes [12].

In MOGAD, pGFAP levels did not differ significantly from healthy controls. During attacks, pGFAP levels were roughly twice as high as in remission but remained lower than those observed in NMOSD relapses. These findings support the role of pGFAP as a marker of astrocytic injury primarily in NMOSD. Elevated pGFAP levels correlated with relapse occurrence and disability (EDSS) in both conditions, indicating a strong association with residual deficits [10].

### 4.3. pNfL/pGFAP Ratio

The median pNfL/pGFAP ratio during remission did not differ significantly between NMOSD and MOGAD patients, likely reflecting low biomarker levels similar to those in healthy controls. During attacks, the markedly higher pGFAP/pNfL ratio in NMOSD supports the concept that astrocyte injury predominates during active disease [1,13]. Chang et al. reported that the sGFAP/sNfL ratio effectively distinguished NMOSD, MS, and MOGAD, with higher ratios being observed during attacks in NMOSD, consistent with more severe astrocyte damage [10].

### 4.4. Biomarkers as Predictors of Disease Activity

Our analyses demonstrated that pGFAP and the pGFAP/pNfL ratio were predictive of disability (EDSS), whereas pNfL alone showed excellent accuracy (AUC = 0.906) in predicting recent relapses within the past six months. The predictive capacity of pGFAP was lower, highlighting pNfL’s potential as a more reliable short-term marker of disease activity.

### 4.5. Clinical Utility and Routine Monitoring

Our findings reinforce the pathophysiological distinctions between MOGAD and NMOSD: plasma GFAP reflects oligodendrocytopathy in MOGAD and astrocytopathy in NMOSD, whereas pNfL indicates secondary neuroaxonal damage in both conditions. The ability of pNfL to predict relapses highlights its potential as a routine biomarker for disease activity, facilitating early intervention. GFAP may serve as an indicator of long-term tissue damage and neurodegeneration. To date, direct comparative studies of blood NfL and GFAP across NMOSD and MOGAD remain limited. Most research has focused on these biomarkers within individual diseases, with few studies performing head-to-head comparisons. Further investigation is needed to elucidate the similarities and differences in biomarker dynamics across these conditions. Given the small patient cohorts, it is essential to interpret our results with caution. Limited sample sizes of both patients and controls may have constrained the ability to detect significant differences or associations. Age and sex may influence NfL and GFAP levels; therefore, studies should apply statistical models that account for these demographic variables. The use of Z-scores adjusted for age and BMI has also been suggested to improve interpretation of NfL results. Almost all patients (97%) in both groups were on immunosuppressive therapy, which could have influenced biomarker levels, but treatment effects were not analyzed in our study. Our results are exploratory; thus, the predictive performance of NfL for relapse activity should be interpreted cautiously, as the small sample size and single-center setting may overestimate the accuracy of these findings, and further research is needed to confirm these associations or trends.

Taken together, our results suggest distinct yet partially overlapping pathophysiological mechanisms in NMOSD and MOGAD. In NMOSD, significantly elevated plasma GFAP levels during relapses reflect primary astrocytic injury, whereas increased pNfL indicates secondary neuroaxonal damage resulting from astrocyte dysfunction [9,10,20]. In MOGAD, the predominant pathology involves oligodendrocyte injury, with moderate astrocytic involvement observed during acute inflammatory attacks [11,21]. Strong correlations between pGFAP and pNfL in both disorders indicate interconnected glial and axonal injury. Overall, these biomarker profiles—GFAP > NfL ratio in NMOSD versus balanced or lower GFAP/NfL ratio in MOGAD—highlight distinct cellular targets while reflecting a shared component of secondary neuroaxonal degeneration, consistent with previous studies [22,23,24].

### 4.6. Limitations of the Study

This study has several limitations that should be considered when interpreting the findings. The small sample size of both patients and controls may have restricted the ability to detect significant differences or associations, and the results should therefore be interpreted with caution. The single-center design may also contribute to an overestimation of the predictive accuracy of observed associations.

Demographic and clinical confounders—including age, sex, comorbidities, BMI, treatment regimens, and lifestyle factors—were not fully controlled and could have influenced plasma NfL and GFAP concentrations. Almost all patients (97%) in both groups received immunosuppressive therapy, which could have influenced biomarker levels; however, the effect of treatment on biomarkers was not analyzed in our study. Future studies with larger cohorts and standardized sampling protocols are warranted to clarify the impact of specific immunosuppressive therapies on NfL and GFAP levels.” Another limitation of our study is the absence of longitudinal analyses of within-patient changes, which would capture individual-level variations in biomarker concentrations over time. Future studies could apply mixed-effects or other longitudinal models to investigate dynamic biomarker changes during both remission and relapses.

Research on NfL and GFAP in MOGAD remains limited, partly due to its recent reclassification as a distinct disorder; prior to 2023, many MOGAD patients were classified as having NMOSD, complicating the understanding of disease-specific biomarker patterns. Comparative studies between NMOSD and MOGAD are scarce, highlighting the need for larger, multicenter, longitudinal studies to validate our observations and establish clinical meaningful thresholds.

Furthermore, the study population was restricted to a single center and a single ethnicity. It is important to note that the cohort lacked ethnic diversity, which may limit the generalizability of our findings to more diverse populations. Long-term follow-up data are lacking; therefore, the present findings should be regarded as exploratory and hypothesis-generating.

## 5. Conclusions

This study supports plasma NfL and GFAP as reliable biomarkers of tissue injury in NMOSD and MOGAD. Elevated NfL reflects ongoing neuroaxonal damage and predicts relapses, whereas increased GFAP in NMOSD indicates astrocyte injury, particularly during attacks. The NfL/GFAP ratio highlights distinct patterns of injury between the two diseases. Despite these findings, direct comparative studies between NMOSD and MOGAD remain limited. Further research is warranted to clarify biomarker differences and enhance disease monitoring.

## Figures and Tables

**Table 1:** Demographic and clinical characteristics.

	NMOSD	MOGAD	HCs	Difference Between NMOSD and MOGAD *SMD* (*p* Value)	Difference Between NMOSD and HC *SMD* (*p* Value)	Difference Between MOGAD and HC *SMD* (*p* Value)
Participants, *n* Female, *n* (%)	18 18 (100)	12 5 (42)	39 26 (66.7)			
Age (years), mean (SD)	49.5 (10)	34.6 (13.2)	39 (12.5)	0.000	0.001	NS
Disease duration, median (IQR), years	10 (3.5–13)	11 (3.7–13)	-	NS	-	-
Time since the last attack, median (IQR), months	14.5 (5–49)	11 (4–22)	-	0.03	-	-
EDSS, median (IQR)	4.0 (2.5 −5.5)	3.5 (2.6–4)	-	0.049	-	-
Number of relapses, median (IQR)	2 (1–7)	3 (2–8)	-	NS	-	-
pNfL, median (IQR), pg/mL	6.9 (4.7–10.2)	6.4 (3.3–10)	5.1 (4–6.9)	NS	0.048	0.018
pNfL in the attack, median (IQR), pg/mL	9 (6.9–37.4)	10.4 (7.3–40.8)		NS	0.000	0.005
pNfL in remission, median (IQR), pg/mL	5.2 (4–8.5)	3.6 (2.7–5.4)		0.034	NS	0.005
pGFAP, median (IQR), pg/mL	169 (136–223)	129 (97–223)	133 (91–144)	NS	0.002	NS
pGFAP in the attack, median (IQR), pg/mL	335 (181–416)	224 (110–297)		NS	0.004	0.018
pGFAP in remission, median (IQR), pg/mL	149 (131–197)	121 (89–140)		NS	NS	NS
pGFAP/pNfL, median (IQR)	28.5 (20–32.5)	24 (12–36)	29 (20–35)	NS	NS	NS
pGFAP/pNfL in the attack, median (IQR)	28 (9–37)	8.3 (4–12)		0.016	NS	0.000
pGFAP/pNfL in remission, median (IQR)	29 (21–32.6)	36 (20–46)		NS	NS	NS

EDSS, expanded disability status scale; NS, not statistically significant; pNfL, plasma neurofilament light chain; pGFAP, plasma glial fibrillary acidic protein; SD, standard deviation; IQR, interquartile range.

**Table 2:** Correlation analyses of variables in a cohort of AQP4-IgG+ NMOSD and MOGAD patients.

Patient Population	AQP4-IgG+ NMOSD (*n* = 18)			MOGAD (*n* = 12)		
Variable	pNfL (pg/mL)	pGFAP (pg/mL)	pGFAP/pNfL Ratio	pNfL (pg/mL)	pGFAP (pg/mL)	pGFAP/pNfL Ratio
Age	0.556 **		−0.676 **	0.596 **		−0.496 *
EDSS	0.582 **	0.601 **	−0.640 **		0.452 **	
Disease duration			0.407 *			
Total number of relapses						0.563 **
Time since the last attack	−0.597 **			−0.513 **		0.790 **
pNFL		0.643 **			0.702 **	
pGFAP	0.643 **			0.702 **		

AQP4, Aquaporin-4; EDSS, expanded disability status scale; MOG, myelin oligodendrocyte glycoprotein; MOGAD, MOG antibody-associated disease; NMOSD, neuromyelitis optica spectrum disorder; pNfL, plasma neurofilament light chain; pGFAP, plasma glial fibrillary acidic protein; * correlation at a significance level of 0.05; ** correlation at a significance level of 0.01.

## Data Availability

The data presented in this study are available upon request from the corresponding author.

## Abbreviations

The following abbreviations are used in this manuscript:

AUC	Area under the curve
AQP4-Ab	Aquaporin-4 antibody
BMI	Body mass index
CI	Confidence Interval
EDSS	Expanded Disability Status Scale
GFAP	Glial fibrillary acidic protein
HCs	Healthy controls
IQR	Interquartile range
IMT	Immunosuppressive treatment
MRI	Magnetic resonance imaging
IPND	International Panel for NMO Diagnosis
MOG	Myelin oligodendrocyte glycoprotein
MOG-Ab	MOG antibody
MOGAD	MOG antibody-associated disease
NMOSD	Neuromyelitis optica spectrum disorder
NfL	Neurofilament light chain
ROC	Receiver operating characteristics
SD	Standard deviation
SMD	Standardized mean differences
SIMOA	Single molecule array
